# Beyond Genes: Re-Identifiability of Proteomic Data and Its Implications for Personalized Medicine

**DOI:** 10.3390/genes10090682

**Published:** 2019-09-05

**Authors:** Kurt Boonen, Kristien Hens, Gerben Menschaert, Geert Baggerman, Dirk Valkenborg, Gokhan Ertaylan

**Affiliations:** 1 VITO Health, Boeretang 200, Mol 2400, Belgium; 2 Centre for Proteomics, University of Antwerpen, Antwerp 2020, Belgium; 3 Department of Philosophy, University of Antwerp, Antwerp 2000 & Institute of Philosophy, KU Leuven, Leuven 3000, Belgium; 4 Biobix, Department of Data Analysis and Mathematical Modelling, Ghent University, Ghent 9000, Belgium; 5 Hasselt University, Hasselt 3500, Belgium

**Keywords:** proteomics, re-identifiability, privacy, genomics, data, personal medicine

## Abstract

The increasing availability of high throughput proteomics data provides us with opportunities as well as posing new ethical challenges regarding data privacy and re-identifiability of participants. Moreover, the fact that proteomics represents a level between the genotype and the phenotype further exacerbates the situation, introducing dilemmas related to publicly available data, anonymization, ownership of information and incidental findings. In this paper, we try to differentiate proteomics from genomics data and cover the ethical challenges related to proteomics data sharing. Finally, we give an overview of the proposed solutions and the outlook for future studies.

## 1. Personal Genomics and Proteomics

Current discussions and regulations regarding high throughput, molecular level (OMICS) data center around two seemingly opposite values. On the one hand, there is the duty of the researcher to protect the research participants’ integrity and privacy, on the other hand, there is a scientific imperative to share data with other researchers. Indeed, the technological and scientific transformation we are experiencing in the era of high throughput OMICS technologies, is partially attributed to the sharing of research data across studies, institutes and international borders. Thus, it has become the norm for many data types generated via genomics and other high-throughput technologies, to make study information widely and effectively accessible. This very fundamental principle of data sharing allows the scientific community to be transparent and the scientific process to be reproducible and accountable. The technological and ethical challenge is that this sharing of data involving human participants must be performed in a way that adequately protects the interests of the participants.

We are living in the era of data economy where personal data (in any form) can be traded, mined, analyzed and used for commercial or other gains. Recent scandals regarding the use of personal data of individuals that were unaware of this (e.g., the Cambridge Analytica case [1]), and the fact that evolving technologies question the idea that genomic data can be truly anonymized, poses a threat to the principle of privacy. Proteomics data is considered relatively safe for sharing traditionally, but today it consists of an ever larger amount of sequence information; this to an extent that is similar to genomic level data, where anonymization and privacy is recognized to be absolutely crucial.

Here, we want to go beyond the issues associated with genetic and genomic research, and demonstrate that in the field of proteomics, a thorough reflection on the aforementioned issues is imminent since precision medicine, relying heavily on various kinds of OMICS data, is rapidly changing the future of diagnostic and therapeutic practices. Although most of the discussions in the public domain are on genomic data, biological entities (like cells, tissues and organs) interact with their environment through proteins, RNAs and metabolites, and not directly via its genome. The genotype is relatively fixed and knowledge of the genotype translates itself into risk percentages for certain diseases that can develop throughout a person’s life. However, proteins (in the form of transcriptomics, proteomics and peptidomics) provide more biologically relevant information on the current state of the phenotype. An in depth analysis of the transcriptome, proteome and metabolome can be regarded as a more precise molecular phenotype (called proteotype in the case of proteins [2]) that can enable detection of diseases and disease states other than solely genetic diseases. Furthermore, gene, protein and metabolite level data analyses can also provide complementary approaches that can be linked across data sources [3] and integrated at the systems level. One example is the usage of clinical biochemistry, proteomics and metabolomics signatures to determine personal disease risk profiles using (combnations of) biomarkers. Another application is pharmacoproteomics, where the effects of drugs on protein levels are monitored [4]. Proteomics also provides crucial knowledge in fundamental research of disease aetiology and can therefore aid the translation of basic research into therapies in a supportive or leading role. Lastly, the information content related to a coding gene increases during its expression and function (due to processing, modifications, cellular location, etc.) and proteomics can provide information on these aspects [5], which is relevant since these modifications are often dysregulated in disease.

Proteomes can also be acquired on the cellular up to the systemic level (organs, blood, urine and cerebrospinal fluid) and can therefore represent their origin, which makes proteomics indispensable in precision medicine. Proteomics is therefore not only a “watered-down” version of genomics privacy wise but in addition contains information on a person’s phenotype. Therefore, it should be treated with care and also presents new idiosyncratic challenges for its use in personalized medicine.

## 2. Proteomics as Sensitive Data

Genomic data is conventionally recognized as strictly personal and it is currently under debate if it even is possible to anonymize it (see further) [6]. However, it is not yet clear to what extent proteomics and metabolomics data require the same status. Metabolites, as an end-product of metabolic pathways, can probably be considered non-personal except for rare metabolic syndromes. This discussion refers to the proteomics characterization by use of mass spectrometry (MS), since this technique relies on peptide sequencing (e.g., instead of the use of antibodies in multiplexed ELISAs), resulting in a representation of the genome. Antibody based chips are usually not directed against peptides with single amino acid variations, unless they are specifically designed to do so. Proteomics is currently treated as non-personal data in the scientific community (e.g., by the National Cancer Institute, US). This opinion is historically based on the fact that, until the advent of the new generation of mass spectrometers (and bioinformatic methods), the depth at which proteomes are charted is limited compared with genomics. However, as mass spectrometers become more sensitive and faster, the percentage of the sequence information that can be obtained through proteomics increases. Additionally, there is a necessity to verify the experimental workflows in proteomics since these are more prone to experimental errors when compared to genomics and open access to the unprocessed data is often required by scientific publishers to preserve the integrity of data quality. The privacy risks in proteomics need to be clearly reviewed and working solutions have to be proposed; e.g., the design of a data format that does not contain sensitive and/or personal proteomic information but still informative enough for research when made available to third parties. Another pertinent question is how much phenotypical information (such as disease/risk status) on a subject is associated with the proteomic profile. Proteomics can therefore be twofold sensitive: It can identify people and provides clues about their health.

A typical proteomics pipeline contains more steps than genomics and the eventual data from proteomics is multilevel. Genomics provides genetic sequences while proteomics can produce partial peptide sequences, peptide identifications, protein identifications (with the corresponding protein coverage, i.e., how much of the protein is covered by peptide identifications), modification profiles, quantification and pathway level information. This can all be extracted from the raw data obtained with a mass spectrometer. We will discuss raw proteomics data and specify the data level and type when necessary. For example, identification strategies that use single amino acid polymorphisms (SAPs) can use raw and unfiltered peptide level data, whereas protein IDs and quantification metrics will not be regarded as identification (but not health) sensitive data.

Allelic variants in the genome can only propagate to the proteomic level if they are in coding regions (disregarding the more complex question on how these alleles can quantitatively influence protein expression) and if they result in an amino acid substitution that is not isobaric (e.g., a leucine to isoleucine conversion cannot be detected in standard proteomic experiments), these are called SAPs [7]. There were roughly 1.1 × 10^6^–1.3 × 10^6^ SAP reported in 2016 [7]. This number is probably much lower in the genome of a person since it contains mutations from cancer genomes that generally cannot be used for identification purposes. Parker et al. estimated that there are more than 35 × 10^3^ non synonymous single nucleotide polymorphisms (nsSNPs) in exosomes with frequencies over 0.8% [8]. Mutations that alter protein splicing and deletions that cause frame shifts can also occur but are less frequent. Proteomic pipelines can identify all these events with or without using matched genomic data (from genomics or transcriptomics); those using it being the more accurate and sensitive. In proteomics, proteins in a sample are usually identified by enzymatically cleaving the protein and analyzing the resulting peptides since peptides are inherently much easier to measure and identify than full-length proteins (this rationale is called bottom-up proteomics). The digestion step results in a highly complex peptide mixture and there is therefore a need to separate the peptides before entry into the mass spectrometer. Liquid chromatography is the most used peptide separation method. The standard proteomics LC–MS method is data-dependent acquisition (DDA), where the masses of the eluting peptides are measured (MS1) and selected for fragmentation one by one. Fragmentation in the MS (called MS/MS or MS2) generates fragmentation spectra that contain sequence information of the peptide. Each fragmentation spectrum is associated with a mass of the intact proteolytic peptide, with a list of masses of the fragments and method related information like retention time (time of elution). These spectra can be queried by a variety of methods and tools to link the fragmentation spectrum to a protein sequence translated from a genome. Protein databases can be cleaved in silico, after which the theoretical mass of the parent peptide and the fragments are calculated (the retention time and fragment ion intensities can also be predicted) and compared with experimental spectra. The selection and isolation of peptides in the MS is a critical step: usually not all peptides can be fragmented due to time constraints, they can be fragmented at sub-optimal points in their elution profile (resulting in MS2 spectra with a low information content) and the isolation is not absolute, resulting in mixed fragmentation spectra. All these factors hamper the clear delineation of the part of the proteome that is “personal”. Data independent acquisition (DIA) aims to overcome some of these problems by systematically selecting the whole mass range of MS1 for fragmentation in cycles [9]. This means that the issues of selection timing and the semi-stochastic nature of selection are no longer a problem, but this comes at the expense of highly convoluted spectra. These fragmentation spectra are a mixture of all peptides eluting at a certain time from the LC in a window that can range from 2 to a few hundred *m/z*. These spectra can be queried by looking for pre-defined transitions (a combination of parent and fragmentation mass or *m/z*) or be deconvoluted and submitted to a DDA type search engine. The latter is usually less sensitive since it does not use prior knowledge.

There are several ways to detect SAPs in proteomics. The sequence of a peptide can be determined directly from its fragmentation spectra without using any other information. This is called de novo sequencing and requires high quality spectra. The full sequence is usually not possible but short tags (called peptide sequence tags, PSTs) can be identified. For pure de novo (de novo can be integrated in database searches), the nsSNP has to be in a region where fragments are detected. Full fragment coverage is not necessary (a mass shift due to a combination of amino acids can still be reliably determined, although the order is unknown in that case) but is beneficial for de novo sequencing. Database searches in general can only identify peptides that are contained in the database (like dbSAP, [7]). The search space of search engines increases with larger databases, and this can hamper efficient identification of SAPs. Open modification (or error tolerant) searches can also be used [10] but suffer from the same problem (unless they are combined with e.g., spectral matching in hybrid approaches [11]). An optimal search space is acquired by using custom transcriptomics or RiboSeq based protein databases [12]. Database searches can identify more peptides than pure de novo approaches since the prior knowledge lowers the amount of evidence needed for a positive identification. However, it is always advisable to systematically double check modifications and mutations if a very large search space is used. DIA systematically searches for fragment ions that are unique for a peptide in a convoluted spectrum. Fragments and transitions that specifically define a SAP have to be included in the search in order to be able to detect them [13].

However, DIA as well as DDA data can be re-queried with databases that contain SAPs or SAP specific transitions. Especially DIA is well suited for re-analysis since it contains in principle fragments of all detectable peptides, including their elution profile. DDA data can also contain “cryptic” fragments in mixed spectra.

DDA and DIA are both screening methods that are not hypothesis driven. Targeted proteomics, where only a limited number of proteins (maximal a few hundred) are measured, is an alternative hypothesis based strategy. For each protein, a few well responding peptides are selected, and from these proteins a few (selected reaction monitoring or SRM) or all (parallel reaction monitoring or PRM) transitions are measured. These methods have inherently a low chance of picking up SAPs, unless they are specifically designed for them [14]. SRM has the lowest sensitive information content, followed by PRM, DDA and DIA.

Several fields in proteomics are currently interested in detecting SAPs. Especially proteogenomics deals with this, where the most developed field is oncoproteogenomics since tumor mutations are of particular interest in fundamental and diagnostic cancer research [15,16,17]. On a practical note, the International Cancer Genome Consortium has established that although it should largely treat the non-cancerous sequencing data as personal data, genetic variants specific to tumor cells are nonetheless anonymous, with only rare exceptions. Hence they freely distribute the anonymous variants to other researchers in accordance with the principle of open science. Another remark is that many nsSNPs that have a physiological effect are altering the protein modification profile (post-translational modifications or PTMs), for example new phosphorylation or ubiquitination sites. PTM specific techniques (phosphoproteomics being the most widely used) can therefore enrich SAPs in the sample. Related to oncoprote(ogen)omics is immunopeptidomics, where the peptides presented by the antigen presenting MHC1 and MHC2 complexes are analyzed. The aim of these studies is often to determine which peptides could possibly be used to train immune cells to combat diseases such as cancer [18]. Of course, the exact peptide sequence is of utmost importance when investigating possible antigens. In oncology, of high interest are the mutation-derived neoantigens released by cancer cells that can initiate an anti-tumor immune response. A third field is the search for proteins that remain undetected by proteomics methods [19,20,21]. There can be several reasons for the lack of proteomic evidence of proteins and sequence variation, including the absence of the correct sequence in the database, is one of them. Evidently, reanalysis of the unidentified spectra can be an efficient strategy to identify SAP containing peptides. Finally, the use of proteomics in forensic sciences is currently being explored and initial reports that try to identify people using proteins in hair, bone and epidermal cells found in fingerprints indicate that the approach is valid [8,22,23,24]. The technical challenges of SAP detection, and validation, in the aforementioned fields are met with increasingly more refined and powerful bioinformatic tools, like spectral libraries [25], specialized databases [26,27,28,29], pipelines for quality control [19,21,30,31], and more recently, fragment intensity prediction software that is incorporated in DDA and DIA search engines [32,33]. SAPs can now be detected and quantified from 100 μg of serum proteins [34], used to discriminate cancer patients from healthy individuals and can be detected in a handful of cells [35].

A person can be identified by the combination of their nsSNPs. The likelihood of a positive identification can be calculated by multiplying the frequencies of the allele corresponding to the nsSNP. Li and colleagues used only the minor allelic frequencies of nsSNPs and adjusted the likelihood of identification by the global peptide false discovery rate [36]. The analysis proved that enough nsSNPs from minor alleles can be detected in serum/plasma (up to 20) for identification, and this minimal requirement of SAPs will soon pose even less of a problem since the performance of LC–MS systems is increasing rapidly. The study also already pointed out some of the peculiarities of proteomics compared to genomics data. First of all, proteomics data is a subsample of the genome and the amount of identifiable data depends on the sample. Second, an additional layer of uncertainty is connected to peptide identifications, as every single peptide identification has a probability value attached to it and highly significant SAP identifications can be weighed more than less significant identifications. DDA, DIA, PRM and SRM all have theoretical frameworks to control the false discovery rate and have methods to assign confidence to a particular identification in an experiment. Third, the bioinformatic identification stage is the main cause of differences in interlaboratory reproducibility (hence the need for submission of data to public repositories). Various software approaches for SAP detection will give different results, and the overlap and differences in results will influence identification likelihoods. For example, results from DDA search engines do not completely overlap and identification results from DIA (when using transition lists) can vary depending on used spectral library. Some of these additional layers of complexity are currently being addressed by the scientific community and soon will open the path towards proteomics as a mature complement of the genomic data which warrants premeditation when sharing and integrating proteomics data with other data repositories.

The General Data Protection Regulation (GDPR) came into effect in 2018 in the European Union. Genetic data is defined in the GDPR as “personal data relating to the inherited or acquired genetic characteristics of a natural person, which result from the analysis of a biological sample from the natural person in question, in particular chromosomal, deoxyribonucleic acid (DNA) or ribonucleic acid (RNA) analysis, or from the analysis of another element enabling equivalent information to be obtained”. Proteins provide equivalent information of genetic data, as discussed above, and as such are also genetic data. It even means that the likelihood of “real world” identification, for example by linking SAPs to genetic data in (formerly) available repositories and having a strong link to an existing person, is not required to classify it as personal data. If the data can theoretically be used for identification purposes, it is considered personal genetic data, even if there are practical challenges that impede a researcher of directly doing so. However, this does not mean that proteomics data cannot be practically anonymized (see further).

Additionally, the GDPR also considers health related data as sensitive. SAPs can give a first look into a person’s health if they are associated with nsSNPs that have a known corresponding genetic risk factor. There is also an increasing list of protein biomarkers for various diseases. These biomarkers are usually measured with ELISAs or related antibody based technologies since absolute and accurate quantification of biomarkers with LC–MS can be a tedious process, but possible nonetheless. For example, a comparison between protein immunoprecipitation MRM (multiple reaction monitoring, a form of targeted proteomics) and ELISA has shown that there is a high correlation between both [37]. For some biomarkers, LC–MS based methods are actually preferred, as in the case of the various forms of amyloid β that are difficult to distinguish with antibodies [38]. There is also a recent trend in LC–MS based quantification where relative and absolute quantifications are combined [39], which could be very useful in biomarker research. Lastly, the predictive status of proteomic profiles is currently being explored: Can we correlate phenotypes with proteomics data across experiments instead of just focusing on one or a small set of proteins? If so, this would indicate that the proteomics profile of clinical matrices could be used to infer the health status of a person. The advent of personalized medicine and the use of proteomics herein, will probably stimulate these developments.

## 3. Ethical Issues in Personal Proteomics Research

The gathering of large amounts of data that are necessary for a systems biology approach to personal medicine creates specific ethical questions. Much has already been written regarding issues related to (genetic) biobanking and genetic research [40,41]. For example, current discussions focus on the right to genetic privacy of research subjects and of patients donating samples for genetic research. Whereas ten years ago, the ethical and legal discussions on genetic privacy focused primarily on whether samples and data should be anonymized, coded or identifiable, and how such biobanks had to be governed [42], today the challenges of whole genome sequencing and the idea that it may be impossible to completely anonymize DNA are discussed [6]. Other discussions center around the right of participants to receive information about their DNA that is used in research, or the duty of researchers or even clinicians to return incidental findings that may or may not be of some clinical utility to the patients or research subjects [43]. In genetic research, ethical frameworks regarding consent (can participants give blanket consent? Is consent needed even for leftover samples?) and solidarity (is strict consent needed if the research benefits society?) were already proposed [44]. The discussion on the ethical aspects of proteomics can be picked up by the current discussion on genomics data and biobanking. We would like to emphasize that it will most likely not be possible to have one answer that would fit all situations (and study conditions) but an internationally recognized set of ethical guidelines to follow up would be a step forward. This would potentially assist the ethical committees advising personalized medicine and clinical proteomics studies.

Anonymization and identifiability with regard to genetic samples is a broadly covered topic, but proteomics may raise some new specific questions. As we described above, it may be possible to obtain DNA information from proteins, which would immediately make all the ethical issues related to genetic research also relevant for proteomics. Moreover, the fact that also phenotypic information may be deduced from proteomics, makes this issue even more pertinent. Indeed, one of the reasons why privacy is important with regard to such information is the possibility that this information is misused by third parties such as employers and insurers. For the purposes that they would be interested in, phenotypic information is more interesting than genotypes alone.

One of the solutions that was proposed to avoid problems related to privacy and confidentiality is complete anonymization of samples. Anonymization is the irreversible alteration of any type of personal data so that its human subjects are no longer identifiable. This process is incompatible with longitudinal follow-up, and is therefore generally discouraged in precision medicine. It is understandable that anonymization is seen as an attractive option to comply with data protection laws. Indeed, the GDPR does not seek to regulate anonymized data, while insisting on keeping data in an identifiable form for no longer than necessary for the purposes for which it is processed.

So far when it comes to genomics level data GDPR links the assessment of identifiability to available technology where all attempts on anonymization fell short by the next available technology. First it was shown by Gymrek and colleagues [45] that it was possible to identify people with their surnames based solely on their DNA and trace amounts of associated metadata. They have concluded that even a few markers from one person can spread through deep genealogical ties and lead to the identification of another person who might have no acquaintance with the person who released their genetic data. Another feature of their identification technique was that it relied entirely on free, publicly available resources, which can be executed with only computational tools and an Internet connection. The identification is proven to be possible even if the amount of information was as small as 25 randomly selected loci from the whole genome [46]. This number of loci can already be achieved by current proteomic technologies, as was discussed, and results from this study can therefore be extrapolated to proteomics. Further complicating the situation is that it was demonstrated to be possible to accurately and robustly determine whether individuals DNA are present in a complex genomic DNA mixture [47]. Although this technology could potentially be very useful in forensic science it hampers the efforts to make the efforts of aggregate genomic data (such as GWAS) publicly available.

Genomic data-sharing beacons provides an easy to implement, standardized and secure solution for genomic level data-sharing by explicitly allowing yes/no queries on the presence of specific alleles in the beacon content. Previously deemed secure against re-identification attacks, beacons are also very recently demonstrated to be vulnerable despite their very stringent policy. Although the risks are not comparable to identification of individuals with their surnames, recent studies have demonstrated that it is possible to determine whether a person is in the dataset, by repeatedly querying the beacon for his/her single-nucleotide polymorphisms (SNPs) [48].

In spite of this situation, we believe it is still premature to conclude that genomic data cannot be anonymized, and a fortiori the impossibility to anonymize proteomic data especially since the end-product of a proteomics pipeline is highly processed. For the moment, it is unsure whether raw proteomics data can be fully anonymized. However, the various levels of proteomics data can provide more opportunities for anonymization than genomics data (see below). We believe therefore that participants in future personalized medicine studies including proteomics should be informed about new evolutions regarding identifiability of proteomics (and genomics) samples themselves. An open communication regarding this issue with research participants is warranted, as this will help build trust. A reason why complete anonymization of samples may be problematic (from an ethical point) is the fact that certain health information that is relevant to the research participants themselves may be discovered. How to deal with these incidental findings has been a hot topic in the debate regarding genomic data and the question will be even more pertinent in proteomics.

All matters become even more complicated when studies integrate various data from genetic testing, clinical markers (epigenetics, proteomics and metabolomics) and state of the art sensors, as is done increasingly to monitor people’s health. A typical use of proteomics in such endeavors is the longitudinal follow up of urine or various types of blood samples. Such varied forms of data can provide insight in (a) health risks related to genetic, environmental and behavioral factors, (b) insights in molecular mechanisms associated with disease and (c) possible leads for new therapies. However, the data obtained also generates even more privacy issues concerning a person’s health. This poses even harder ethical, practical and legal challenges. It will be possible to determine personal health risks (for instance based on an individual’s genetic profile) while, at the same time, monitor changes in molecular pathways associated with these health risks. Although this opens up the possibility to go to a system of personalized prevention, it also creates an urgent need to establish an ethical framework for such studies that will try to tackle problems associated with the use of these technologies and the results they will produce. In these cases, a framework needs to be established in which participants can be informed on actions to improve health or prevent disease. An important part of this ethical framework will pertain reporting (non-)incidental findings and risk factors. Increased knowledge of one’s health can increase the mental burden of being responsible for one’s own health, but risk factors and lifestyle recommendations should be balanced by the best knowledge on their effectiveness.

## 4. Consent

Issues related to data privacy, and the return of results, should be clearly communicated in consent forms. Asking consent from research participants should not solely be seen as a means to protect against legal claims, but also as a means to generate and maintain openness about the research, and hence to enable trust in research. As such, it is an acknowledgement of the fact that participants may have an opinion on what kind of research is done, and that their opinion matters. The scope, scale and duration of large-scale proteomics studies requires the development of new technologies, technological implementations and ethical recommendations concerning consent of people for collecting, analyzing and sharing their data. The medium (e.g., electronic) and clear language of the consent is very important. GDPR states that broad consent has to be avoided but repeated requests for approval can lead to “consent-fatigue”. In addition, due to cohort-size, sampling frequency and data types and density, acquiring specific consent for follow-up or additional studies can soon become very complicated. A workable solution, legal, technical and ethical, of obtaining additional consent from participants for new analysis has to be worked out. An example is dynamic consent, using an electronic platform that minimizes the effort of both asking and receiving consent to a minimum. We envision that different consent mechanisms for academic research and economic valorization will be required. In the case of economic valorization, reward mechanisms for the participating individuals (if target participant numbers are reached) could be coupled to form a feedback mechanism that will result in a sustainable ecosystem.

## 5. Current Precautions and Possible Working Solutions

The amount of personal health data is increasing at a fast pace and the value of such aggregated data has been recognized by commercial entities (such as Google’s Verily Life Sciences, Mountain View, CA, USA; 23andMe, Mountain View, CA, USA; Nebula Genomics, San Francisco, CA, USA; PatientsLikeMe, Cambridge, MA, USA, etc.). The last few years it has become apparent that there are serious risks in terms of privacy and data safety in giving commercial companies unrestricted access to not anonymized personal data. However, efficient use of integrated personal data can greatly benefit both general health (including healthcare and pharmaceutical industry) and personal health. One could make the claim that since the individual is the source and the owner of personal data, if used in association with the individual identified, (s)he should also directly benefit from sharing personal health data. Development of such future frameworks in personalized medicine would require dynamic consent and coupled to rewarding mechanisms. These frameworks are already starting to be developed in commercial companies that analyze genomic data. New standards on personal data management need to be developed or implemented for these frameworks to fully mature. The participant’s privacy needs to be safeguarded at all times, ensuring that true data ownership is with the participant. Methodologies, knowledge and the aggregated (anonymized) data by itself can be used for economic valorization. A business model that is fair to the participants is strongly preferred (third party commercial access is reviewed on a case per case basis), and policies will have to be put in place to ensure that the data shared with third parties cannot be re-identified or abused in other ways.

An important factor in the discussion is the ability to achieve true anonymization of health data, proteomics included. In this scenario, the aggregated data would be a highly valuable economical commodity whose ownership would be less obvious and traded and used more easily. The challenges ahead are to determine the definition of sensitive and identifiable information within the proteomics datasets and whether this information can be removed or made inaccessible without significantly reducing the scientific quality.

The various levels of data in proteomics entail various levels of data sensitivity. Health related data is only sensitive if it can be linked to a person. Fortunately, health related data can be largely dissociated from peptide level data that can identify a person, so a first step in determining the possibility of anonymization would be to delineate the research question and to determine which level of proteomics data is needed and sufficient to answer it. Typical research questions can be:

- Is a protein detected in a dataset?
- Is the protein identification reliable?
- Is the PTM profile different between conditions?
- Are there different pathways upregulated between conditions?
- Is the statistical analysis supported by the data? For example, is the proposed biomarker indeed a good candidate to discriminate healthy persons from diseased?

The main reason for open access in clinical proteomics and its application in personalized medicine is the reliability of the bioinformatics workflows. This pertains the protein identification but, in the case of quantitative proteomics also normalization and transformation of the data. Quantitative data extraction can easily be done on known non-SAPs, having a reliable feature detection algorithm being the only liability. Processed quantitative data can therefore be disseminated without privacy risks. Likewise, looking for proteins in online datasets and PTM analyses can be processed and filtered to leave out SAPs. This means that only analyses directly involving detecting and reporting SAPs in raw data should be scrutinized in assessing re-identifiability issues. Working towards consensus identification and quantification pipelines would address parts of this problem.

Genetic data is not available to the public because of the obvious privacy reasons. The same is true for genetic information in proteogenomics. The translated database used for querying, as well as the identified SAPs can pose a risk. The only truly anonymous raw proteomics data is SRM, if the measured transitions are not defined by an nsSNP. The security risk in PRM is present but can be evaluated if there are SAP identifying transitions in the retention time and mass range of the peptide. For DDA and DIA, the absence of privacy risks in raw data might never be fully guaranteed. One solution proposed [36] was processing the raw data by removing fragmentation spectra of SAPs. This is a reasonable solution, but not a conclusive one since there still can be fragmentation spectra from SAPs that escaped detection due to software or, more probably, database issues. One could argue that the remaining SAPs are too few and/or of too poor quality to conclusively pose identification risks, but this remains to be investigated. Critselis E. recently proposed to submit batches of pseudonymized data instead of individual data [49], but this would only work if the relation between the SAPs is removed completely. This means that MS/MS spectra of all experiments would be submitted as a whole (so processed data, not raw data), and the identifications and accompanying FDR estimations are done experiment instead of sample wise. In this setup, the identification step and the quantification step can be separated, but both could be made publicly available in an altered format that is anonymous. Of course, the latter two proposals are DDA specific, since safely publishing sensitive DIA would require even more processing.

Another option would be to implement search engines in proteomics repositories, and researchers interested in investigating a privacy sensitive data set would perform their analyses on a server, where after the results are filtered for SAPs. The availability of raw data on servers that is accessible to other proteomics researchers after permission would not alter the original purpose of the research (for which consent was given) and would be in the public interest since the reliability of proteomics research is reviewed and monitored continuously. There are currently national computer clusters that are dedicated to sensitive data and allow a federated analysis, like the Swedish Bianca cluster. Sweden also has a local version of the European Genome–Phemome Archive (EGA-SE), which has restricted access; e.g., as used in [50]. Open repositories could also have different access levels that allow us to query all the data except for the sensitive. A filter for meta-analyses of open repositories would also be an option. The reason for open repositories are stated above, and a stringent reviewing process of clinical LC–MS data analyses by multiple third parties, as an additional qualitative part of paper submission and reviewing, would increase the confidence in the conclusion of clinical proteomics and partially counter the main reason for submission to open repositories. The practical feasibility of these options, and other not proposed by the authors, will have to be discussed by the proteomics community, since the last and least attractive option would be closed repositories.

Personalized medicine, with its various data formats (genomics, proteomics, metabolomics, etc.), is a more challenging domain. Recently blockchain technology is being proposed [51,52] as a distributed electronic ledger for hosting health information. Blockchain technology allows us to create a distributed, transparent, independent and secure private information ledger where health data providers (individuals) are in control, own their information and can monitor access privileges as well as being informed about who accessed their information. Although, this technology is new in the health domain and currently only realized by a single start-up (Genomes.io) [53,54], it has potential for growth since it enables a data-driven marketplace to be created where users can receive tangible benefits for making their data accessible and immutable to the research organizations, application development community, pharmaceutical and consumer businesses. Blockchain alone does not solve the re-identifiability problem, however it addresses the issue of consent while simplifying and incentivizing data sharing in a secure and transparent manner. Currently the market leader is the KSI^®^ blockchain technology stack developed by Guardtime, which is being used by NATO, the US Department of Defense, Lockheed Martin, Boeing, Ericsson, Telstra, SAP, GE and in Estonia where majority of the state data systems utilize the blockchain technology to enforce the integrity of government data and systems. The application of blockchain technology for personalized medicine (e.g., by the Estonian Genome Center, Tartu, Estonia) is currently being implemented.

## 6. Conclusions

A consistent increase in both depth (resolution) and size (population range) of proteomics studies not only allows significant scientific progress but also poses ethical challenges regarding personal privacy sharing for where reproducibility is a fundamental pillar in science, the fact that it may be possible to deduce genomic information from proteomic data means discussions regarding privacy, sharing of results and regulations that are applicable to genomics may also become relevant for the proteomics field (not all researchers might be aware of this currently). Moreover, the fact that proteomics represents a level between the genotype and the phenotype may introduce even more intricate questions related to data access, ownership of information and incidental findings. We believe that trust in research is a precious commodity to be protected. This means, on the one hand, that researchers should be made aware of these potential issues. Researchers should know that existing privacy regulations might also apply to proteomics research and be conscious about this when dealing with proteomics data. On the other hand, research participants have the right to know about these issues, both about what we know now and what we may know in the future. Consequently, consent procedures may need to be revised from time to time and platforms for dynamic consent may be set up to allow for smooth communication between researcher and participants.

Therefore we envision the next generation of scientific enterprise is a highly collaborative environment where researchers recognize that they are entrusted with invaluable personal information, and research participants feel their data is safe, where demands of open science and the need for data protection are consolidated.
